# Corticosteroids or platelet-rich plasma injections for rotator cuff tendinopathy: a randomized clinical trial study

**DOI:** 10.1186/s13018-021-02470-x

**Published:** 2021-05-21

**Authors:** Haleh Dadgostar, Farinaz Fahimipour, Alireza Pahlevan Sabagh, Peyman Arasteh, Mohammad Razi

**Affiliations:** 1 Sports Medicine Department, Rasoul Akram Hospital, Iran University of Medical Sciences, Tehran, Iran; 2 Department of Orthopedic Surgery, Rasoul Akram Hospital, Iran University of Medical Sciences, Tehran, Iran; 3 Shiraz University of Medical Sciences, Shiraz, Iran

**Keywords:** Corticosteroid, Platelet-rich plasma, Rotator cuff, Tendinopathy, Supraspinatus

## Abstract

**Background:**

Studies evaluating the role of both corticosteroids and platelet-rich plasma (PRP) in the treatment of rotator cuff (RC) tendinopathies have been contradicting.

We compared structural and clinical changes in RC muscles after corticosteroids and PRP injections.

**Methods:**

This is a randomized double-blind clinical trial. All individuals with diagnosis of RC tendinitis during 20142017 were considered. Individuals were randomly allocated to either receive PRP or corticosteroids.

Overall, 3cc of PRP was injected within the subacromial joint and another 3cc was injected at the site of the tendon tear, under the guide of sonography. For the corticosteroid group, 1cc of Depo-medrol 40mg and 1cc of lidocaine (2%) was injected within the subacromial joint.

**Results:**

Overall, 58 patients entered the study. Comparison of pain, range of motion (ROM), Western Ontario RC (WORC), Disability of Arm-Hand-Shoulder (DASH) scores, and supraspinatus thickness showed significant improvement during follow-ups in both groups (*p*<0.05).

During 3 months of follow-up, pain improvement was significantly better within the PRP group during (from 6.662.26 to 3.082.14 and 5.531.80 to 3.881.99, respectively; *p*=0.023). Regarding ROM, the PRP group had significant improvement in adduction (20.508.23 to 283.61 and 23.217.09 to 28.464.18 for the PRP and corticosteroid groups, respectively; *p*=0.011) and external rotation (59.6623.81 to 76.6618.30 and 57.1424.69 to 65.5726.39, for the PRP and corticosteroid groups, respectively; *p*=0.036) compared to the corticosteroid group.

**Conclusion:**

We found that PRP renders similar results to that of corticosteroids in most clinical aspects among patients with RC tendinopathies; however, pain and ROM may show more significant improvement with the use of PRP. Considering that the use of corticosteroids may be contraindicated in some patients and may be associated with the risk of tendon rupture, we suggest the use of PRP in place of corticosteroid-based injections among patients with RC tendinopathy.

**Trial registration:**

Clinical trial registration code: IRCT201302174251N9

## Introduction

Shoulder pain is among the most common complaints in medicine and disorders attributed to the RC are the most common cause of shoulder pains [1]. More than 50% of all shoulder pains are considered to be that related to tendinopathies of the RC, as supraspinatus incomplete thickness tears and tendinosis [2].

In clinical assessment of RC tendinopathies, rehabilitation time can be lengthy as the healing ability of tendons is limited; thus, treatment modalities have been introduced that focus on the potential to induce more rapid healing. Conventionally, physical rehabilitation, rest, and nonsteroidal anti-inflammatory drugs (NSAIDs) are considered for a patient with RC tendinopathy as the first line of treatment [3, 4]. If the patient does not respond to these, the more commonly used treatment modality for patients are sub-acromial injections of corticosteroids, which have been shown to be affective in accelerating the healing process. Corticosteroids have been more effective during the acute phase of tendinitis, although they have been associated with risks of tendon tear and may inhibit collagen synthesis [5, 6].

More recently, platelet-rich plasma (PRP) has been recognized for its possible biological role by providing cellular and humeral mediators which may improve the healing process [7, 8].

Studies have been conducted in recent years evaluating the role of both corticosteroids and PRP in the treatment of tendinopathies related to the RC; however, these studies have been somewhat contradicting as some have found no difference between placebo controls and PRP and corticosteroids and some have reported more rapid benefits with PRP injections among patients [2, 8,10]. Moreover, almost all the aforementioned studies have mainly focused on factors such as pain and functional outcomes, so here, we aimed to evaluate the structural changes in the muscles of the RC after corticosteroids in comparison to PRP injections in the settings of a double blind clinical trial study.

## Patients and methods

### Study settings and patients

This is a randomized double-blind clinical trial conducted in the Hazrat Rasoul Hospital, Tehran, Iran. All individuals who had a complaint of shoulder pain during January 2014 to January 2017 were initially considered for the study. Individuals who had tendinitis or incomplete tear of the RC tendons, which was confirmed with MRI, had pain for more than 3 months, were older than 40 years old, and had a total of three positive tests out of the following five tests: neer, speed, full can, empty can, and the Hawkins test, were included in the study.

Those who were unwilling to enter the study or did not refer for follow-ups, those who did not follow the study protocol, had radicular pain, had signs of other pathologies including frozen shoulders or calcified tendinitis, those who had complete tear of RC tendons, had surgery during the past 6 months, had inflammatory diseases such as rheumatoid arthritis, fibromyalgia, polymyalgia rheumatica, and etc., had ligamentous laxity (a positive apprehension test or sulcus test), and those who had corticosteroid-based injections in the shoulder during the past 3 months, were excluded from the study.

These individuals were randomly selected to enter the study using simple random selection.

### Randomization

Individuals were classified into two groups of PRP and corticosteroid groups, using the permuted block randomization method. All randomizations were done by the Department of Sports Medicine at Iran University of Medical Sciences, Tehran, Iran.

### Study design

During visit at related clinics, complete medical history on pain and specific data on functional problems of the shoulder were acquired. After which complete examination of range of motion of shoulder, specific tests related to tendon function within the RC and shoulder biceps were performed.

For evaluating the RC, each patient underwent MRI of the shoulder for confirmation of tendinopathy of shoulder. Tendinitis was considered when signal of the tendon changed while tendon was preserved. Incomplete tear of the tendon was considered a detachment of the tendon without including the whole thickness of the tendon.

Patients were then referred for ultrasonography. Tendons of the shoulder were evaluated by a specialist in sports medicine. Sonography was done using a Mindray M5 system (China) and by a 7L4s transducer.

All assessments were done by a single sports medicine specialist who was blinded to the grouping of patients, in order to minimize bias in measurements.

### Definition of variables

For subjective evaluation of patients, the Western Ontario RC (WORC) and Disability of Arm-Hand-Shoulder (DASH) questionnaires were utilized. After detailed explanation, each individual filled in the questionnaires.

The WORC questionnaire is a measure of functional limitations in RC pathology. This index considers 5 variables in its assessment, including physical symptoms, physical activity, work, daily activity, and mentality and excitement. Each item is scored from 0 to 100, and the higher the score the worse the condition of the shoulder [11].

The DASH questionnaire is a measure of disability and includes 30 questions which assess individuals ability to perform daily activity, for example: ability to carry objects, writing, and other daily activities. Patients are scored from 0 to 100 and a higher score indicates a worse condition [12].

Pain was assessed using the Visual Analogue Scale (VAS) questionnaire. In this system of scoring, a picture is shown to the individual which is consequently scored from 0 to 10. The higher the number, the higher the severity of the pain [13].

Range of motion of shoulder was directly evaluated by a manual medical Goniometer (Dahi Teb, Iran) [14]. Range of motion (ROM) of shoulder, abduction, adduction, forward flexion, internal and external rotation, and extension were assessed according to the Kendall method [13]. Measurement of ROM was done by one trained expert in sports medicine. To reduce bias, all ROM tests were performed three times and mean of these measurement were considered for each individual.

Data on baseline characteristics, range of motion, WORC score, DASH score, and VAS score was obtained from each patient. The minimal detectable change (MDC) for the WORC, DASH, and VAS is reported to be 1.7 [15], 10.2 [16], and 0.08 [17], respectively, according to previous literature.

### Intervention

The PRP was prepared and processed in ROOYA GEN (Arya Mabna Tashkhis Co., Tehran, Iran). For the corticosteroid injections, the Depo-medrol 40mg (Pfizer Inc., USA) was used.

For the administration of PRP or corticosteroid, patients were seated. After proper preparation, 3cc of PRP was injected within the intraarticular joint and another 3cc of PRP was injected at the site of the tendon tear, under guide of sonography [2].

For the corticosteroid group, 1cc of Depo-medrol 40mg and 1cc of lidocaine (2%) was injected within the subacromial joint similar to the PRP group.

After the injections, patients were asked to withdraw from using any non-steroidal anti-inflammatory drugs (NSAID). In cases with pain, they were allowed to use acetaminophen.

After the injections, both groups were given shoulder exercise and scapula dyskinesia regimens. Patients were blinded to the treatment groups and were unaware of the type of injections received.

### Follow-ups

Baseline information, medical history, and physical examination of patients were recorded during first visit. Patients were then visited 1 week, 1 month, and 3 months after administration of medication. In cases of pain or any complaint patient were able to either contact or refer to the sports clinic.

DASH and WORC scores were evaluated at baseline, 1 month, and 3 months post-intervention; however, other parameters including ROM and VAS were evaluated at five occasions of baseline, 1 week, 1 month, and 3 months post-intervention.

Thickness of the supraspinatus muscle was evaluated at baseline, 1 month and 3 months of follow-up.

### Allocation concealment

All follow-ups were done by a single specialist in sports medicine who was blinded to the allocation of patients. A clinical trial nurse was appointed to remove any information from patients medical records prior to visitation by the assessor.

### Primary endpoint

Pain using the VAS score.

### Secondary end points

(1) Range of motion, (2) supraspinatus thickness, (3) WORC score, and (4) DASH score

### Sample size calculation

Severity of pain, evaluated using the VAS questionnaire, was considered the primary outcome of the study. Correlation of VAS from beginning of the study to the end point was 0.4 (*r* = 0.4). For obtaining a statistical difference of 13 or more, and considering a standard deviation of 20 [13], type one error of 5% and a power of study of 80%, a minimum of 30 individuals were needed for each of the study groups.

### Ethical consideration

All participants gave their written and informed consent to take part in the study. The study protocol was in coherence with the guidelines of the Declaration of Helsinki.

### Statistical analysis

Data was analyzed using the SPSS software, for windows, version 23. All dependent variables were tested by Shapiro-Wilk test for normality. The assumption of normality was accepted when *p* value was >0.05. For comparison of qualitative data between groups, the chi-sqaure test and for comparison of quantitative data with normal distribution between two groups the independent *t* test was used. For comparison of scores during follow-ups, the ANOVA test (GLM Repeated measures test) was used.

A *p* value of less than 0.05 was considered statistically significant.

## Results

In total, 58 patients entered the study. Two patients were lost to follow-up after 3 months in the corticosteroid group.

Comparison of baseline characteristics and baseline clinical assessment including ROM, WORC score, DASH score, and supraspinatus thickness in US is shown in Table1.

**Table 1:** Baseline and clinical characteristics of the PRP and corticosteroid groups

Variables	Groups			
	PRP (***n***=30)	Corticosteroid (***n*** = 28)	Overall	***p*** value
Ageyears	57.33 9.80	53.60 7.24	55.53 8.79	0.108
Sexno. (%)				0.644
Male	5 (16.7)	6 (21.4)	11 (19)	
Female	25 (83.3)	22 (78.6)	47 (81)	
VAS score	6.66 2.26	5.53 1.80	6.14 2.15	0.041
Flexiondegrees	116.90 37.58	135.42 35.70	126 38.15	0.060
Extensiondegrees	35.16 10.78	41.42 10.87	38.21 10.19	0.017
Abductiondegrees	102.83 36.07	118.46 41.43	109.32 39.50	0.130
Adductiondegrees	20.50 8.23	23.21 7.09	22.05 7.73	0.186
Internal rotationdegrees	64.26 17.06	60.17 19.41	62.73 18.26	0.397
External rotationdegrees	59.66 23.81	57.14 24.69	58.39 24.49	0.694
WORC score	32.85 19.43	35.56 17.97	34.46 18.81	0.585
DASH score	54.02 18.24	52.50 20.32	53.51 1925	0.764
Supraspinatus thicknessmm	6.97 1.46	7.47 1.38	7.20 1.43	0.354

*PRP* platelet-rich plasma, *VAS* Visual Analogue Scale, *WORC* Western Ontario Rotator Cuff, *DASH* Disability of Arm-Hand-Shoulder

Results showed that regarding baseline ROM assessments, the PRP group had a more limited ROM in extension (35.16 10.78 vs. 41.42 10.87, *p* = 0.017). Moreover, the PRP group also showed higher degree of pain in baseline assessments compared to the corticosteroid group (Table1).

Comparison of pain, ROM, WORC, and DASH scores and muscle thickness showed significant improvement during follow-ups in both groups (*p* < 0.05).

Comparison of the two groups showed that changes in pain was significantly higher within the PRP group compared to the corticosteroid group during the 3-month follow-up period (from 6.66 2.26 to 3.08 2.14 and 5.53 1.80 to 3.88 1.99, respectively; *p* = 0.023). Regarding ROM, the PRP group showed more significant improvement in adduction (20.50 8.23 to 28 3.61 and 23.21 7.09 to 28.46 4.18 for the PRP and corticosteroid groups, respectively; *p* = 0.011) and external rotation (59.66 23.81 to 76.66 18.30 and 57.14 24.69 to 65.57 26.39, for the PRP and corticosteroid groups, respectively; *p* = 0.036) compared to the corticosteroid group.

The two groups did not show any difference in improvement of other measures of ROM, WORC score, DASH score, and supraspinatus thickness during the follow-up period (*p* > 0.05) (Table2).

**Table 2:** Comparison of PRP and corticosteroid groups during a 3-month follow-up.

Variables	Groups										
	PRP				Corticosteroid				***p*** value	Fisher value	Effect size
	Baseline	1st week	1st Month	3rd Month	Baseline	1st week	1st Month	3rd Month			
VAS**	6.66 2.26	5.36 1.89	3.75 2.15	3.08 2.14	5.53 1.80	4.86 1.99	3.84 2.07	3.88 1.99	0.023	3.868	0.670
Flexiondegrees**	116.90 37.58	118.40 40.03	132.73 39.06	139.50 34.6	135.42 35.70	139.42 36.80	140.09 36.98	148.65 34.5	0.112	2.326	0.041
Extensiondegrees**	35.16 10.78	37.50 10.40	46.16 11.19	50.33 9.18	41.42 10.87	42.69 13.05	50.76 9.02	52.50 8.15	0.302	1.220	0.022
Abductiondegrees**	102.83 36.07	107.43 37.19	115.66 36.75	141.83 36.04	118.46 41.43	119.61 41.10	127.50 43.68	138.26 40.24	0.081	2.572	0.045
Adductiondegrees**	20.50 8.23	21.16 8.37	26.50 4.57	28 3.61	23.21 7.09	26.53 6.28	27.69 4.73	28.46 4.18	0.011	5.087	0.086
Internal rotationdegrees**	64.26 17.06	68.50 17.12	78.50 13.3	82.16 10.39	60.17 19.41	63.84 22.64	77.50 15.18	79.61 13.55	0.741	0.333	0.006
External rotationdegrees**	59.66 23.81	65.83 23.12	74.83 20.36	76.66 18.30	57.14 24.69	60.19 26.81	64.03 26.42	65.57 26.39	0.036	3.475	0.060
WORC score**	32.85 19.43		40.66 18.76	49.93 22.36	35.56 17.97		44.87 21.59	48.46 20.60	0.315	1.166	0.021
DASH score**	54.02 18.24		48.83 13.53	40.83 18.19	52.50 20.32		44.77 17.89	41.05 15.69	0.520	0.658	0.012
Supra-spinatus thicknessmm*	6.97 1.46			6.36 1.06	7.47 1.38			7.40 1.07	0.119	2.509	0.044

*PRP* platelet-rich plasma, *VAS* Visual Analogue Scale, *WORC* Western Ontario Rotator Cuff, *DASH* Disability of Arm-Hand-Shoulder, *Effect size* Eta squared

*p = 0.051, **p < 0.001, these two represent difference and changes during follow-up in the PRP and corticosteroid groups separately

## Discussion

In this study, we compared the results of two of the most common treatment methods, PRP and corticosteroid injections, for those with tendinitis or incomplete tear of RC tendons of the shoulder, in the settings of a double blind clinical trial study. To the best of the authors knowledge, this is the first time that ultrasonography structural changes have been considered for assessment after a combination of exercise and injection of PRP or corticosteroids.

We found that those who received PRP injections showed significant improvement in pain after three months of follow-up compared to the corticosteroid group. Regarding ROM, those who received PRP also showed better improvement in adduction and external rotation during our follow-up period.

Regarding muscle thickness, evaluated using ultrasonography, the two groups did not show any difference in any of the follow-ups.

Despite biological justification for the clinical efficacy of PRP use in the settings of tendinopathy, high level evidence on the effects of PRP in tendinopathy is scarce [18,20]. In a study by Scarpone et al. [21] evaluating the effects of PRP among patient with RC tendinopathy resistant to physical rehabilitation and corticosteroids, they found that after injection of zilocain (1%) and 3.5ml of PRP within the lesion, during a 52-week follow-up, functional scores showed a significant improvement (at weeks 8 and 12 of follow-up). Moreover, VAS score also showed significant improvement at 12 and 52 weeks of follow-up. Regarding ROM, they found the best improvement in external rotation at 12 weeks of follow-up (*p* < 0.001).

Another study by Kesikburun et al. [9] compared the effects of PRP injections with that of saline injection (placebo) in a clinical trial study. They included a total of 40 patients with tendinopathies of the RC and or with partial tear of the RC. Participants had sub-acromial injections of PRP under the guide of sonography. They compared the two groups regarding WORC index, pain (VAS), and quality of life, and they found no difference between the two groups regarding any of the variables during 1-year follow-ups. This study showed the ineffectiveness of PRP compared to a placebo control. This was attributed to multiple factors including the effects of exercise regimens and the needling processes which itself stimulates bleeding and the consequent tissue and tendon repair.

On the other hand, another study by Wesner et al. [22] found that patients receiving PRP showed better function and reduced pain compared to a group that received placebo during a 6-month follow-up. Moreover, they also found that those who received PRP had better improvement of RC in MRI findings, although those in the placebo group did not show any significant change.

Von Wehren et al. [23] also compared autologous conditioned plasma or ACP with the effects of corticosteroids among patients with partial symptomatic tear of RC in 2016. They compared the two groups regarding VAS, Simple Shoulder Test (SST), The American Shoulder and Elbow Surgeons Shoulder Score (ASES), and the Constant-Murley Score (CMS). In their study, similar to ours, they found that the ACP group (compared to the corticosteroids group) had a better ASES, VAS, and CMS after 12 weeks of follow-up compared to the baseline. They did not find a significant difference between the two groups in MRI findings.

In a study by Shams and colleagues in 2016 [2], they compared the effects of PRP injections compared to that of corticosteroid injections among patients with incomplete tear of RC tendons in the settings of a clinical trial study. They found that after 6 months of follow-up, those who receive PRP injections report less pain. However, they did not find a statistically significant difference between the two groups regarding function and MRI findings. We did find a significant improvement in pain, although unlike the mentioned study, we did record a significant improvement in range of motion after 3 months of follow-up in the PRP group.

Our study showed that both PRP and corticosteroid injections can alter and improve function of the shoulder in RC tendinopathies. However, during our 3-month follow-up, the PRP group showed better improvement in VAS index which is a specific measure of pain and in ROM.

Although we should consider that our study showed that most benefits of PRP compared to corticosteroids in earlier follow-ups (3 months in our study), and perhaps longer follow-ups, minimize the benefits of PRP injections over corticosteroid injections, considering that the ROM is more affected by exercise rather than injections of PRP or corticosteroids. Our findings are similar to that of the latter study as they concluded that perhaps ACPs show their effects earlier than that of corticosteroids.

One of the interesting findings in our study was that we did not document any change in the thickness of the supraspinatus muscle over time in any of the groups. This shows that perhaps improvement of function in the shoulder is not necessarily associated with improvement in imaging and paraclinical tests, and further studies with longer follow-ups are needed to evaluate the relationship between paraclinical findings and functional improvement after intra articular injections.

This study was not without limitation. As this was a clinical trial study, some individuals did not refer for their follow-ups and were lost during the study. Our study did not have a placebo control, which may have affected our final results, although the main objective of our study was to compare two of the most common injectable medications used for tendinopathies. We only had a single session of PRP injections, although some studies have advised the injection of a second dose of PRP one to 2 months from the initial injection of PRP [9]. In our study, our corticosteroid group also received one session of injection to increase homogeneity between groups.

Considering that some paraclinical changes including muscle thickness may require more than 6 months to show significant change and improvement, our follow-up visit may have been short. Moreover, MRI provides a more accurate tool for assessment of structural changes.

As the supraspinatous muscle is the most common muscle involved in RC tendinopathy [24], we focused on changes on this specific muscle.

Considering the limitations of current literature, we propose future clinical trials to include a large sample size and to consider factors such as the use of placebo control and more injection sessions and even the use of combination of medications as one recent study [8] showed that the simultaneous use of both corticosteroids and PRP may be possible as the two medications do not interact.

## Conclusion

We found that PRP renders similar results to that of corticosteroids in most clinical aspects among patients with RC tendinopathies; however, pain and ROM may show more significant improvement with the use of PRP. Considering that the use of corticosteroids may be contraindicated in some patients and may be associated with the risk of tendon rupture, we suggest the use of PRP in place of corticosteroid based injections among patients with RC tendinopathy.

## Data Availability

All authors and institutions can access the raw data by directly contacting the corresponding author at Farinaz.fahimipour@gmail.com.

## Abbreviations

PRP: Platelet-rich plasma
RC: Rotator cuff
ROM: Range of motion
WORC: Western Ontario RC
DASH: Disability of Arm-Hand-Shoulder
NSAIDs: Nonsteroidal anti-inflammatory drugs
VAS: Visual Analogue Scale
SST: Simple Shoulder Test
ASES: American Shoulder and Elbow Surgeons Shoulder Score
CMS: Constant-Murley Score
